# Methods for Establishing a Renal Cell Carcinoma Tumor Spheroid Model With Immune Infiltration for Immunotherapeutic Studies

**DOI:** 10.3389/fonc.2022.898732

**Published:** 2022-07-28

**Authors:** Leonard Lugand, Guillaume Mestrallet, Rebecca Laboureur, Clement Dumont, Fatiha Bouhidel, Malika Djouadou, Alexandra Masson-Lecomte, Francois Desgrandchamps, Stephane Culine, Edgardo D. Carosella, Nathalie Rouas-Freiss, Joel LeMaoult

**Affiliations:** ^1^ Hemato-Immunology Research Department, CEA, DRF-Francois Jacob Institute, Saint-Louis Hospital, Paris, France - U976 HIPI Unit, IRSL, Paris University, Paris, France; ^2^ Department of Medical Oncology, Saint-Louis Hospital, AP-HP.Nord - Université de Paris, Paris, France; ^3^ Service d’Anatomopathologie Saint-Louis Hospital, AP-HP.Nord - Université de Paris, Laboratory of Pathology, UMR-S728, Paris, France; ^4^ Department of Urology, Saint-Louis Hospital, AP-HP.Nord - Université de Paris, Paris, France

**Keywords:** spheroid, renal cell carcinoma, immune checkpoint inhibitors, standardization, PD-1, heterogeneity, personalized medicine

## Abstract

Tumor spheroids play an increasingly important role in cancer research. Their ability to recapitulate crucial features of tumor biology that are lost in the classically used 2D models along with their relative simplicity and handiness have made them the most studied 3D tumor model. Their application as a theranostic tool or as a means to study tumor-host interaction is now well-established in various cancers. However, their use in the field of Renal Cell Carcinoma (RCC) remains very limited. The aim of this work is to present methods to implement a basic RCC spheroid model. These methods cover the steps from RCC tumor dissociation to spheroid infiltration by immune cells. We present a protocol for RCC dissociation using Liberase TM and introduce a culture medium containing Epithelial Growth Factor and Hydrocortisone allowing for faster growth of RCC primary cells. We show that the liquid overlay technique allows for the formation of spheroids from cell lines and from primary cultures. We present a method using morphological criteria to select a homogeneous spheroid population based on a Fiji macro. We then show that spheroids can be infiltrated by PBMCs after activation with OKT3 or IL-15. Finally, we provide an example of application by implementing an immune spheroid killing assay allowing observing increased spheroid destruction after treatment with PD-1 inhibitors. Thus the straightforward methods presented here allow for efficient spheroid formation for a simple RCC 3D model that can be standardized and infused with immune cells to study immunotherapies.

## Introduction

Advanced clear cell renal cell carcinoma (ccRCC) is primarily treated with systemic treatments targeting the tumor microenvironment (TME) These treatments include immune checkpoint inhibitors (mainly PD-1 inhibitors) or VEGFR tyrosine kinase inhibitors (TKIs), as single agents or as part of combination treatments depending mostly on patient’s prognosis. To this day, apart from the clinical IMDC prognostic criteria, which are associated with decreased sensitivity to VEGFR-TKIs, no reliable biomarkers are available in the clinic to select the best therapeutic options for each patient. The use of standardized patient-derived individual predictive models may help solve this unmet clinical need, as well as serve in the translational research setting.

Current ccRCC models regroup *in vivo* and *in vitro* models, each with advantages and disadvantages. While *in vivo* models provide the possibility to study tumor biology in a dynamic and more complete TME, they come with cost, poor handiness, and ethical limitations. Furthermore, their application as a theranostic tool, mostly in the form of patient-derived xenografts (PDXs) remains far remote from clinical routine. Thus, relevant *in vitro* ccRCC models are essential both as research tools and as theranostic tools. As a research tool, while established cell lines grown in monolayers are cost-effective, easy to maintain and allow high replicability and standardization, they include neither the clonal heterogeneity nor the rich TME that are characteristic of ccRCC (1). Conversely, primary cultures of tumor cells directly isolated from patient samples may retain some heterogeneity (including the conservation of several tumor clones as well as TME components) and allow patient-level applications. Two-dimensional culture of either cell lines or primary cultures were successfully used for decades to select, grow and study tumor cells. However, 2D models find their limit when tumor heterogeneity and structural characteristics are key parameters, because those are completely lost (2). These characteristics include tumor physical structure, the impact of hypoxia or the contribution of stromal cells and immune infiltrate to tumor biology. Furthermore, by using 2D models, even for primary culture, a selection still occurs. It is not based on the capability to escape immune destruction or survive and grow in a chemically inadequate environment, but on the cell’s capability to grow attached to plastic in an artificial medium. This has important consequences, among which (i) loss of cell diversity, to the point to which a primary culture becomes a « cell line », and (ii) alteration of the tumor cell characteristics due to culture conditions. In particular, it should be expected that tumor cell characteristics dependent on parameters of the TME may be impacted. For instance, neo-expression of the immune checkpoint HLA-G, which is common in ccRCC cells and is an immune escape mechanism, is usually lost in culture within a few passages (3).

To best preserve tumor structural components, it is possible to work with tissue slices (4–9). However, this model is not suited for studies focusing on individual tumor elements that are heterogeneously distributed within the tumor. In particular, it was shown that immune checkpoints ligands and receptors are heterogeneously expressed in RCC tumors (10). Thus, if focusing on such heterogeneously distributed parameters, tissue slices might not be a good model, being only representative of themselves.

Multicellular tumor spheroids are an intermediate model between 2D cultures and tissue slices, in which tumor components are dissociated and then reassembled in a spheroid of defined parameters. In such spheroids, tumor structure is lost, but they may include tumor cells from different tumor areas along with stromal cells, be of various sizes, possess a necrotic core or not (11, 12) and be infused with immune cells, including autologous Peripheral Blood Mononuclear Cells (PBMCs) and Tumor-Infiltrating Lymphocytes (TILs) (13). Thus, tumor spheroids have the advantages of flexibility, standardization, and parallelization while preserving some tumor heterogeneity. As such, this model seems well adapted to test the effects of immune therapies, as it was shown for colorectal cancer (14–16), breast cancer (17), non-small cell lung cancer (18–20), or liver cancer (21) for instance.

It is clear that such models would be very useful in the context of ccRCC as well, but methodological information on how to generate a basic spheroid model infiltrated with immune cells starting from ccRCC tissue is scarce. Providing such information was our goal here: we present our approach for establishing an immune-infiltrated ccRCC spheroid model. It includes tumor dissociation, primary cell culture, and generation of multicellular tumor spheroids from cell lines or primary cells, and infiltration by immune cells. We also provide a method to select a homogeneous population of spheroids based on morphologic criteria to increase the relevance of the data obtained in the spheroid model. Lastly, we present one application of our model by analyzing the effect of blocking PD-1 with therapeutic antibodies on tumor cell survival in a live-imaging spheroid-killing assay.

## Materials and Methods

All specific materials used are summarized in **Supplementary Table 1** with references. A complete protocol can be found in **Supplementary Data Sheet 1**.

### Human Tissue and Cells

#### RCC Cell Line

The RCC7 cell line originated from ccRCC was kindly provided by Dr. Anne Caignard, St Louis Hospital, Paris (22).

#### Tumor Samples

Human ccRCC tissues were collected as surgical waste from adult patients undergoing nephrectomy at our center (Urology Department, Saint-Louis Hospital, Paris, France), after informed consent. Tumor samples were selected on nephrectomy specimens by a pathologist, rinsed with phosphate-buffered saline (PBS), and placed in Roswell Parks Memorial Institute (RPMI) 1640 medium (Sigma-Aldrich) at 4°C prior to dissociation.

#### Cell Culture

In this study, we describe a custom culture medium suitable for ccRCC cell culture, referred to as RCCM. It is composed of Dulbecco’s Modified Eagle Medium (DMEM, Gibco) and Ham’s F12 media (Gibco) (v/v, 3/1 mixture) supplemented with 10% fetal calf serum (FCS, Sigma-Aldrich), 2 mM L-glutamine (Gibco), 100 U/ml penicillin/streptomycin (Gibco), 10 ng/ml epidermal growth factor (EGF) (Sigma-Aldrich) and 0.4 μg/ml hydrocortisone (Sigma-Aldrich)

Cultures were kept in a humidified atmosphere with 5% (v/v) CO2 at 37°C. Cell transfer and preparation of single-cell suspensions from monolayers were performed by mild enzymatic dissociation using a 0.05% (w/v) trypsin and 0.02% (w/v) EDTA solution in PBS (Gibco).

### PBMC Preparation

Blood samples were collected from healthy donors from Etablissement Français du Sang (Paris). Peripheral blood mononuclear cells (PBMCs) were isolated using gradient Ficoll (Histopaque, Sigma-Aldrich) according to the manufacturer’s recommendations and stored at −150°C.

### ccRCC Dissociation

Within 30 minutes after nephrectomy, ccRCC samples were rinsed in RPMI 1640 medium at 4°C. Fibrous parts were discarded and the remaining was finely minced until reduced to approximately 1mm^3^ pieces.

We chose to avoid further mechanical dissociation as it leads to poor final viability.

For enzyme comparison, the minced samples were incubated in 15mL conical tubes containing 6mL of dissociating solution composed of Hank’s Balanced Salt Solution and 20µL recombinant DNase I (Sigma-Aldrich) with either Liberase TM (Sigma-Aldrich) at concentrations ranging from 0.07 to 0.28 WU/mL or collagenase IV (Worthington) at concentrations ranging from 0.25 to 1 mg.mL-1 at 37°C, in a hot-water bath, for 10 to 60 minutes. Every 10 minutes, the tubes were hand-shaken to help dissociation. No vortexing or repeated up-and-down-pipetting was involved to avoid damaging ccRCC cells. Once the dissociation complete, the solution was filtered through a 100µm nylon cell-strainer and collected in a 50mL conical tube. To rinse the filter and stop the dissociation, 35mL of RPMI 1640 at 4°C supplemented with 10% FCS (Sigma-Aldrich), L-glutamine (300 μg/mL), gentamicin (50 μg/mL) and amphotericin B (2.5 μg/mL, Gibco), referred to as complete RPMI, were poured on top of the strainer and collected in the same tube. Cells were then washed and red blood cells were lysed using red blood cell lysis solution (Miltenyi Biotec) according to the manufacturer’s instructions. After another washing step, cells were resuspended in DMEM and counted. Viability was assessed by trypan blue exclusion. CD45+ immune infiltrating cells and non-immune cells were then separated using CD45 magnetic beads on a QuadroMACS Separator (Miltenyi Biotec) according to the manufacturer’s instructions. Both fractions were collected, washed, and counted. ccRCC patient-derived primary cell cultures were initiated by seeding 10^6^ cells of the CD45-negative fraction in 75cm² flasks. The remaining part of the primary cells was frozen.

After optimization, 0.28 WU.mL^-1^ Liberase TM during 30 to 40 minutes were chosen for dissociation. A complete protocol is provided in **Supplementary Data Sheet 1**.

### Optimization of Culture Medium

ccRCC tumor cells were cultured in a medium constituted of DMEM and Ham’s F12 media (Gibco) (v/v, 3/1 mixture), 10% fetal calf serum (Sigma), 2 mM L-glutamine (Gibco), and 100 U/ml penicillin/streptomycin (Gibco). When indicated, 10 ng/ml epidermal growth factor (EGF) (Sigma-Aldrich), 5 μg/ml insulin (Sigma-Aldrich), 0.4 μg/ml hydrocortisone (Sigma-Aldrich) or 180 μM adenine (Sigma-Aldrich) were added in the medium. Medium was renewed thrice a week.

For growth data collection, tumor cells were seeded at 1,000 cells/cm² and sub-cultured every week. All cultures were performed in flasks. Upon each passage, once per week, numbers of population doublings (PD) achieved by cultures were calculated as follows: PDs = log(N/N0)/log(2), where N0 represents the number of seeded cells and N represents the number of cells obtained after 7 days of growth.

For cell confluence data, confluence was calculated with the Incucyte S2 system (Sartorius) using the dedicated function. ccRCC cells from 3 patients were seeded at 500 cells per well in a 96-well plate and placed in the Incucyte for brightfield imaging every 4 hours for 7 days. Cell confluence was calculated automatically.

### Spheroid Formation

When primary ccRCC cultures or established cell lines reached 80 percent cell-confluence, they were processed for spheroid formation, using the following methods.

#### Liquid Overlay Technique

Cells were detached with trypsin, washed, and counted. They were then diluted with RCCM to reach 50,000 cells/mL. 200µL of this suspension were distributed in each well of a cell-repellent 96-well round bottom plate (Greiner Bio-one). The plate was centrifuged at 500g for 1 minute to initiate contact between cells. Spheroid formation was monitored each day on an inverted microscope.

#### Hanging-Drop Technique:

After detachment, 25µL of a 40,000 cells/mL unicellular suspension of the chosen cells in RCCM were seeded in droplets in the lid of a petri dish. The bottom of the dish was covered in PBS to prevent evaporation and the lid was placed on the dish resulting in hanging drops. Spheroid formation was monitored each day on an upright microscope.

### Spheroid Morphological Parameters Calculation

70 RCC7 spheroids were formed with the hanging-drop technique and imaged every day for five days using brightfield imaging. Obtained images were analyzed using Fiji software (23) for Area, Circularity = (4π×Area)/(Perimeter)², Roundness = 4×Area/(π×major axis²) and Solidity = Area/(convex hull) measurements using a recorded macro (**Supplementary Data Sheet 2**). This macro was used through the plugin>macro>run option on Fiji, it requires the “shape-smoothing” package. The macro improves contrast, uses a threshold to select the spheroid, operates a smoothing of the selection’s border, saves the selection mask in the output folder and measures circularity, roundness, area and solidity along with other shape descriptors. The results are saved in a table at the end of macro execution.

### Spheroid Infiltration

#### PBMC Preparation

To avoid too many washing steps causing cell loss and optimize flow-cytometry results from spheroids, PBMCs were stained for 20 minutes at room temperature with a CD45 APC antibody (eBioscience). PBMCs were then washed and counted. For activation, 30,000 healthy donors PBMCs were seeded in the wells of a 96-well round-bottom plate in 200µL complete RPMI 1640 medium containing 40 ng/mL IL-15 (Miltenyi Biotec) or 100 ng/mL OKT3 (Biolegend) and cultured for 2 days.

#### Monocyte-Derived Cell Preparation

To isolate and differentiate macrophages from human PBMCs, 20 millions healthy donor PBMCs were plated on a standard 100 mm petri dish in RPMI medium. Cells were left 2 hours for adherence and the petri dish was then thoroughly rinsed 4 times using PBS to remove non-adherent cells. The remaining cells were cultured for 3 days in RPMI medium completed with human AB serum (Corning).

#### Spheroid Infiltration With PBMCs

Two days prior to coculture, PBMCs were prepared as described above and 10,000-cell RCC7 spheroids were formed with the liquid overlay technique in RCCM. After these 48 hours, the medium surrounding the spheroids was carefully removed with a 200µL micropipette. CD45-labeled activated or non-activated PBMCs were then added to the spheroids and cocultured for 24 hours. At the end of coculture, the wells were carefully mixed by up and down pipetting without aspirating the spheroid. The supernatants of the resuspended wells were removed, leaving the spheroid in the bottom. Spheroids were then washed twice in PBS following the same process so as to remove all non-infiltrated PBMCs. They were then transferred to 1,5mL Eppendorf tubes, pooling 3 spheroids per tube, and washed once more. PBS was then replaced with 50µL Accutase (Stemcell Technologies) to dissociate the spheroids. Every 10 minutes each well underwent 10 up and down pipetting to combine mechanical and enzymatic dissociation as described by Grässer et al. (24). After 30 minutes of dissociation at room temperature, 450µL of complete RPMI at 4°C were added and the dissociated spheroids + infiltrating PBMCs were analyzed on an Attune NxT flow cytometer (ThermoFisher).

#### Spheroid Infiltration With Monocyte-Derived Cells

10,000-cell RCC7 spheroids were formed with the liquid overlay technique in RCCM were prepared 2 days prior to coculture. 3 days prior to coculture, macrophages were prepared as described above. After these 72 hours, the medium surrounding the spheroids was carefully removed with a 200µL micropipette. Macrophages were detached from the petri dish using trypsin and 30,000 macrophages were added to the spheroids-containing wells. After 24 hours of coculture, the spheroids were processed as described before for washing and dissociation. The obtained single cells suspension was then stained usingCD14-APC (Miltenyi) and anti HLA-DR-PE (Miltenyi) mouse anti human antibodies. Results were analyzed on an Attune NxT flow cytometer (ThermoFisher)

### Spheroid Killing Assay

As described above, 48h before the experiment, 10,000-cell RCC7 spheroids were formed using the liquid overlay technique in RCCM and 100,000 PBMCs were seeded in the wells of a 96-well round-bottom tissue culture plate and activated with IL-15 for 48h. Then, 10 µg/mL nivolumab (Bristol Myers Squibb), 10 µg/mL pembrolizumab (Merck) or no treatment were added to the wells containing PBMCs and incubated for 20 minutes. Cocultures were then initiated: the medium surrounding spheroids was removed and activated PBMCs, non-activated PBMCs or no PBMCs were added to the wells at a 10:1 effector:target ratio. 5 µg/mL of Propidium Iodide (PI) were added to monitor cell death by red fluorescence.

Spheroids were monitored using the Incucyte S2 system (Sartorius), taking 4× images (0.2 NA) with red (585/635 nm excitation/filter) and green (460/524 nm) fluorescence every 2 hours for 5 days. Background red fluorescence was corrected by setting the Green into Red setting at 60 percent so that the control spheroids cultured without PBMCs showed no signs of red fluorescence. Incucyte analysis settings were set with the “spheroid” mode, with the Background to Cell option set to 100 towards background and a minimum object size limit set at “10,000”, allowing selecting only the spheroids. Red fluorescence was analyzed with the option “Largest brightfield object red mean intensity”.

### Statistical Analysis

For ccRCC growth data, analyses were performed with GraphPad Prism 7 (GraphPad Software), statistical significance was tested using paired t-tests.

Other analyses were performed with R (version 4.0.5). Comparisons between categorical and numerical variables for infiltration data were performed using t-tests. For morphological criteria, comparisons between timepoints were performed using paired Wilcoxon-test comparing each group against all (base mean), a global Kruskal-Wallis test was performed, denoting if at least one group is different from the others.

p-values were adjusted for multiple when appropriate using Bonferroni correction, a p-value threshold of 0.05 was chosen for significance. Values are written as mean ± SEM.

## Results

### ccRCC Dissociation

In order to obtain patient-derived RCC cells, dissociation of fresh tumor samples is necessary. Reliable dissociation protocols based on collagenase IV or collagenase cocktails such as Liberase are published, even for ccRCC tumors (25), and even compared (26–28). We will not present data for the optimization of this part, but since the dissociation step is an integral part of the process we present, we felt necessary to individualize this step in a paragraph. Thus, in this study, we used Liberase TM at 0.28 WU/mL for 30 to 40 minutes (**Supplementary Table 1**), which routinely yielded up to 80% viable cancer cells (data not shown).

### Optimization of Culture Medium for Survival, Short-Term and Long-Term Growth of ccRCC Primary Cells

**Figure 1:**
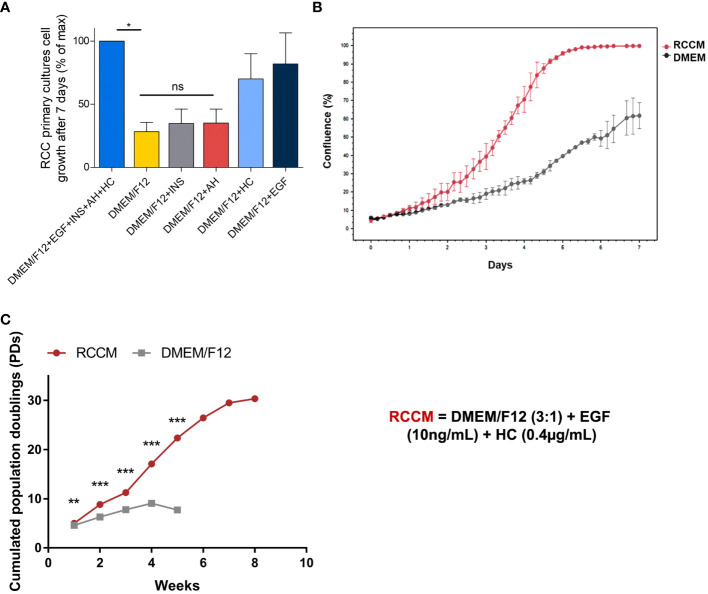
Optimization of RCC cell growth with cytokines. Dissociated cells from ccRCC tumors from 3 patients were amplified in culture media containing the indicated cytokines. EGF: Epithelial Growth Factor, INS: insulin, AH: adenin, HC: hydrocortisone. RCCM: medium supplemented with EGF and HC. **(A)** 7-day proliferation index of ccRCC cells from 3 patients in media supplemented with the indicated cytokines. Maximum proliferation was obtained with the 4 cytokines added to the medium and constituted the 100% mark. All other proliferation results were indexed on this mark. **(B)** RCCM permits faster growth. Confluence % obtained with of primary ccRCC cells over 7 days in RCCM, compared with DMEM/F12 medium alone for one representative patient Confluence % was measured every 4 hours with the Incucyte S2 system and raw values are plotted. **(C)** RCCM permits long-term growth. Long-term FDTR ccRCC cell growth from one selected patient in RCCM or in DMEM/F12 medium alone were compared over an 8-week period. The numbers of population doublings (PDs) attained by cultures were calculated and are plotted. *p < 0.05; **p < 0.01; ***p < 0.001; ns, non-significant.

It is common knowledge that ccRCC primary cells do not survive well in basic culture media such as RPMI and DMEM, and even if obtaining tumor lines by using just those is possible, it is highly inefficient, yielding to death of the entire primary culture in most cases, to weak growth in some, and very rarely in long term growth. Out of commercially available and published primary line culture media we tested, a keratinocyte primary line culture medium proved most reliable. This culture medium is published elsewhere (29) and is composed of base medium (DMEM/F12), i.e. DMEM and Ham’s F12 media (Gibco) (*v*/*v*, 3/1 mixture), 10% fetal calf serum (Hyclone), supplemented with the following components: 10ng/mL epidermal growth factor (EGF) (Sigma-Aldrich), 5 μg/mL transferrin (Sigma-Aldrich), 5 μg/mL insulin (INS) (Sigma-Aldrich), 0.4μg/mL hydrocortisone (HC) (Sigma-Aldrich), 180μM adenine (AH) (Sigma-Aldrich), 2mM tri-iodothyronine (TIT) (Sigma-Aldrich), 2mM L-glutamine (Gibco), and 100U/mL penicillin/streptomycin (Gibco). From this base, we sought to establish a simplified medium suitable for primary culture of ccRCC cells. Tumors from 3 patients were used for this. First, we compared primary ccRCC cell growth when cultured in complete keratinocyte culture medium or in medium with one component removed (**Supplementary Figure 1**). As shown the removal of only 4 of the medium’s components had a negative effect on cell growth: EGF, HC, INS, and AH. Next, we compared the growth of 3 primary ccRCC lines in DMEM/F12 medium alone, supplemented individually with each component, or with the 4 components together (**Figure 1**). As shown in **Figure 1A**, after 7 days of culture, only EGF and HC permitted a significant increase in cell growth compared to DMEM/F12 alone, whereas no effect was observed for INS or AH.

To determine the kinetics of cell growth, we measured cell confluence every 4 hours with the IncuCyte S2 system for one week. We observed that cells showed accelerated growth in DMEM/F12 medium supplemented with EGF and HC compared with DMEM/F12 (**Figure 1B**), yielding to 100% confluence in 5 days, while only 40% confluence was obtained after 7 days in unsupplemented DMEM/F12 medium. This medium will be referred to as RCCM (Renal Cell Carcinoma Medium).

We then determined if EGF and HC could sustain long-term proliferation of primary ccRCC cells. Cells from three patients were amplified in DMEM/F12 or in DMEM/F12 supplemented with EGF and Hydrocortisone (RCCM). Obtaining sufficient amplification in unsupplemented DMEM/F12 became difficult starting from week 4 whereas cells could be amplified for more than 8 weeks in RCCM. Cells amplified in RCCM also showed growth rates twice as high as in unsupplemented DMEM/F12 after 4 weeks (17 population doublings (PDs) vs 9 PDs, respectively), and 30 PDs were obtained after 8 weeks (**Figure 1C**).

RCCM (DMEM/F12+EGF+HC) is therefore a simple medium that reliably sustains survival and growth of tumor cells obtained from dissociated ccRCC samples.

Taken together, our selected methods for ccRCC tumor dissociation followed by culture in RCCM routinely allow us to successfully obtain primary ccRCC cell cultures from 9 out of 10 tumor samples.

### Spheroid Formation Methods From Both ccRCC Cell Lines And ccRCC Primary Cell Cultures

**Figure 2:**
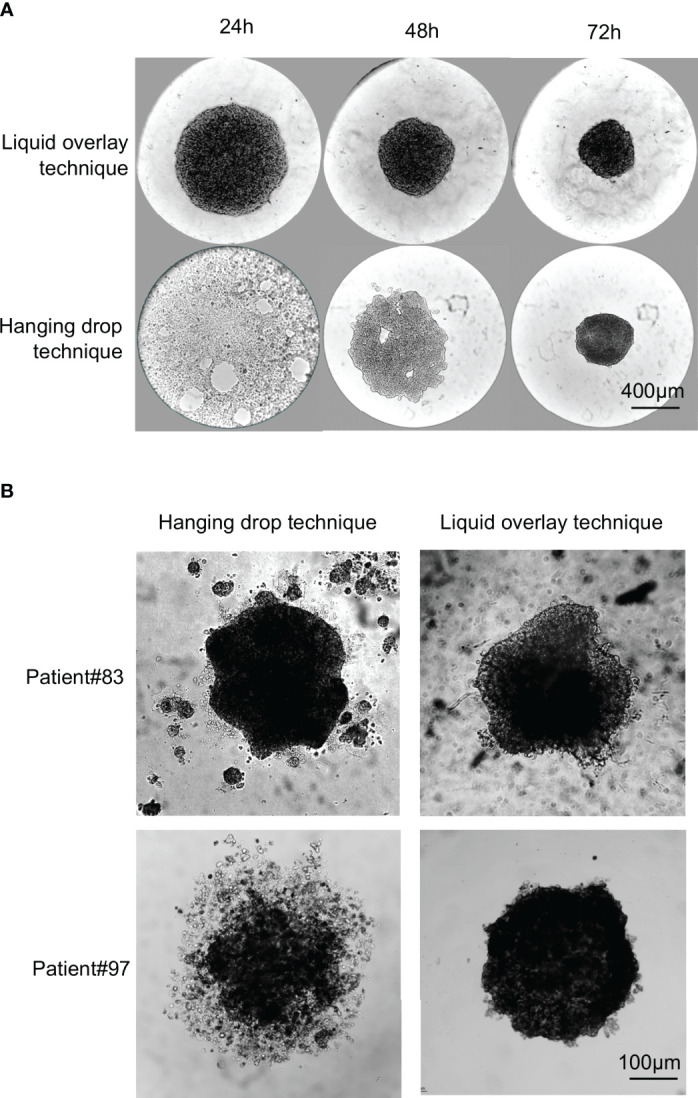
Comparison of spheroid formation from established long-term cell lines and primary lines using the hanging drop or liquid overlay techniques. 10,000 cells from RCC7 cell line or from primary cultures were seeded in droplets and left hanging from the lid of a petri dish (Hanging drop technique) or in the wells of a 96-wells cell-repellent plate (Liquid overlay technique). **(A)** Representative brightfield images of RCC7 spheroid formation using the hanging drop technique or the liquid overlay technique taken every 24h for 72h post-seeding. **(B)** Representative brightfield images of spheroid formation from primary cultures after 72h of culture using the hanging drop technique or the liquid overlay technique.

Depending on the research topic, spheroids may be required either from cell lines or from primary lines, or even directly from dissociated ccRCC cells. Because the successful generation of spheroids directly with ccRCC cells is extremely patient-dependent, we will only present data obtained with established cell lines and primary lines (1 to 2 *in vitro* passages). For the reassembly of spheroids starting from heterogeneous dissociated ccRCC tumor cells, two main methods are described: the hanging drop technique and the liquid overlay technique. In the hanging drop technique, spheroids are formed within droplets of culture medium hanging from the lid of a petri dish, and no specific material is required. In the liquid overlay technique, cells are seeded in wells of commercially available non-stick plates, specially coated to prevent cell adhesion. In both these methods, spheroids form because cells have only each other to adhere to. We compared the formation of spheroids by these two methods by seeding 10,000 cells in droplets or in 96 well cell-repellent plates and by imaging the spheroids in formation every day for 3 days. **Figure 2** shows the results obtained for an established cell line, RCC7 (**Figure 2A**), and for two primary ccRCC cell cultures dissociated from ccRCC tumors and cultured for 1 passage following the procedures presented above (**Figure 2B**).

For the RCC7 cell line (**Figure 2A**), we found that spheroids could easily be formed using both techniques, but that spheroids formed faster (within 24 hours) with the liquid overlay method than with the hanging drop technique (72 hours).

Whereas both techniques seemed suitable for the formation of cell line spheroids of comparable quality, this was not the case with primary ccRCC cultures. We compared spheroid formation by both methods using primary ccRCC cell cultures from 7 patients. Cells from one patient did not form spheroids by either method. Of the remaining 6 patients, results were either similar by both methods (3 out of 6 patients), or better with the liquid overlay method (3 out of 6 patients). **Figure 2B** shows representative results obtained with 1 patient of each category. As can be seen, primary cells from Patient#83 formed spheroids in 72 hours by either method, with no obvious difference observed in spheroids. On the contrary, primary cells from patient#97 formed more spherical and compact spheroids in 72 hours by the liquid overlay than by the hanging drop method.

Thus, in our hands, when working with freshly dissociated cells and primary lines, the liquid overlay method was more reliable and more user-friendly. The spheroids formed by this technique also seemed more regular in shape as described before (30).

### Selection of a Homogeneous Spheroid Population, Based on Automatic Analysis of Morphological Criteria

As a simple 3D model, spheroids are expected to provide an intermediate level between *in vivo* models and cells grown in monolayers. Thus, many authors see them as a “semi high-throughput model” enabling the collection of biologically relevant results with substantial statistical force and relative ease of use. As such, there is a need to optimize the production and analysis of spheroids to reach even higher-throughput and more relevant results. Morphological variation among the tested population of spheroids is known to be a source of inconstancy in the obtained results. This is particularly important in patient-derived spheroids, often subjected to more variability in shape and size than spheroids from established cell lines. Two solutions are possible to mitigate the variations due to spheroid heterogeneity: conducting experiments on a population large enough to reach significant statistical power, or limiting the population heterogeneity itself. For the latter, our approach consists in selecting a homogeneous population, based on morphological criteria. It has been designed to be simple and fast and requires no specific material or software.

To monitor spheroid heterogeneity, 10,000 cell RCC7 spheroids (N=70) were formed with the hanging drop technique, and imaged by simple brightfield every day for 4 days starting on day 4 after cell seeding. The obtained images were automatically analyzed with a recorded Fiji macro (see **Supplementary Data Sheet 2**) calculating the Area of the spheroid along with three shape descriptors: Circularity, Roundness and Solidity. Circularity indicates the degree of similarity to a perfect circle, a value of 1 indicating a perfect circle. Solidity describes the extent to which a shape is convex or concave, a value of 1 indicating a completely convex shape. Roundness is somewhat similar to Circularity but less sensitive to irregularities along the perimeter and more sensitive to elongated shapes (31).

**Figure 3:**
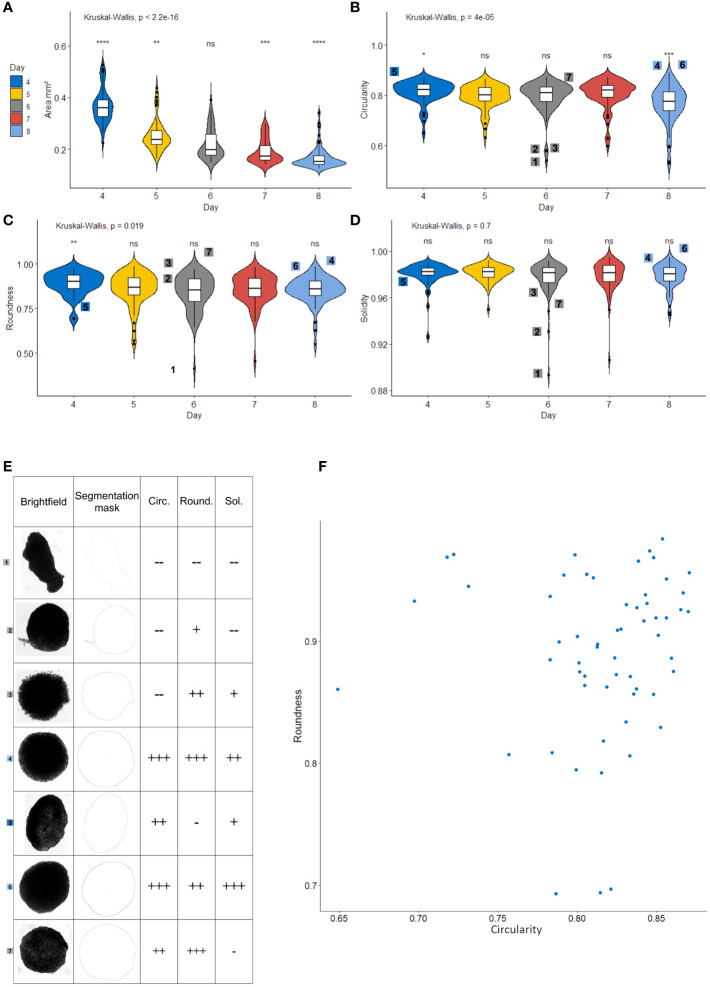
Area, Circularity and Roundness are relevant morphological parameters for selecting a homogeneous spheroid population. Measurement of area **(A)**, circularity **(B)**, roundness **(C)** and solidity **(D)** of Renal Cell Carcinoma cell line Spheroids (N=70) with a recorded Fiji macro. Violin-plots show box-and-whisker plots (white) representing the 1st and 4th quartiles, the line in the middle denotes the median. Whiskers notify the min and max values. The violin part represents the density plot for each group. Digits on the plot relate to the spheroids shown in **(E)**, global Kruskal-Wallis test is given above the chart and pairwise Wilcoxon-Mann-Whitney tests represented above each group, comparing the group to every other (base). **(E)** Illustrative spheroids for roundness, solidity and circularity. Brightfield images, ImageJ masks and levels of measured parameters (–, -, +, ++, and +++). Each of these spheroids is identified with a digit on other graphs. **(F)** Roundness and circularity scatter-plot of the entire spheroid population. all parameters calculated with Fiji software. *p < 0.05; **p < 0.01; ***p < 0.001; ****p < 0.0001; ns, non-significant.

**Figure 3A** shows that the Area of the spheroids formed in this experiment decreased gradually from day 4 to day 8, indicating compaction. Several studies have used area as an indicator of the size of spheroids in terms of growth but we show here that in a model such as ours with short culture times, growth is negligible compared to compaction. Therefore, in short term experiments with little cell growth, area is to be used as an indicator of compactness and not of growth. We observed no significant variation in circularity, roundness and solidity from day 4 to day 8, (**Figures 3B–D**) indicating that these shape descriptors do not correlate with compactness. Consequently, when selecting spheroids for subsequent experiments, both area as an indicator of size/compactness and shape descriptors have to be taken into account.

To better explain the usefulness of each shape descriptors, a set of particular spheroids is shown in **Figure 3E**. First, it is worth noting that abnormal spheroids such as spheroid number 1 present poor scores in each shape descriptor and would therefore not be included in a subsequent experiment. As shown with spheroid 1 and 5, poor roundness is characteristic of elongated spheroids. Spheroids 2 and 7 show that poor solidity is linked to empty spaces or protuberances on the spheroid’s side. Spheroids 2 and 3 link poor circularity to irregularities on the perimeter. In regard to spheroid selection before an experiment, eliminating spheroids 1, 2, 3, and 5 would be preferable. Indeed, spheroid number 1 is unlikely to produce reproducible results, spheroid number 2 presents an abnormal protuberance, spheroid 3 seems to be disintegrating and the elongation on spheroid 5 would lead to an increased surface exposed to the medium during the experiment. On the other hand, it appears that solidity correlates with circularity except for spheroid number 7, which presents a poor solidity due to slightly concave parts along the perimeter. However, this spheroid does not present any particularities that would justify its elimination from the selection. Therefore, we chose not to use solidity in our selection process.

This experiment shows that even when formed from an established cell line, the population of spheroids presents a substantial level of heterogeneity in shape. The scatter plot on **Figure 3F** illustrates this heterogeneity in circularity and roundness on day 4. For subsequent experiments, spheroids should be selected from the population with higher scores for both circularity and roundness.

Altogether, these results suggest that area, circularity, and roundness are relevant quality criteria to select homogeneous spheroids for functional experiments. Priority should be given to Area, as it is an indicator of size and compactness that dictates the surface exposed to the medium as well as the ease of penetration for treatments or immune cells, and then to circularity and roundness. These criteria are of interest mostly when when the number of spheroids for each condition of an experiment is limited by technical issues. Otherwise, increasing the number of spheroids included in the analysis may also be an option.

### Immune Infiltration of RCC Spheroids

The obtained tumor spheroids offer a simple 3D model suitable for many cancer studies. When formed from established cell lines, they contain only cancer cells, making them suitable for studies on cancer cells themselves, such as response to chemical treatments. Heterotypic tumor spheroids such as those obtained from primary cultures or directly after tumor dissociation contain not only a more heterogeneous population of cancer cells, but may also include other cells, such as stromal cells. This makes them suitable for studies involving interactions between cancer cells and other elements of the tumor mass. However, for studies involving interactions between cancer cells and an immune component, immune cells must be introduced and infiltrate the spheroids. These cells can be allogeneic or autologous Peripheral Blood Mononuclear Cells (PBMCs) or even the very Tumor Infiltrating Lymphocytes (TILs) that were extracted from the tumor that formed the spheroid. Thus, we will describe one method to infiltrate spheroids with allogeneic PBMCs and we will illustrate the use of such infiltrated spheroids in the context of checkpoint blockade with therapeutic anti-PD-1 antibodies.

In order for PBMCs lymphocytes to significantly infiltrate spheroids, activation is necessary. This activation can be T-Cell Receptor (TCR)-dependent, using anti-CD3 (OKT3) for instance, or TCR-independent, with IL-15 for example. TCR-dependent activation can be used when TCR function is not required in downstream experiments, and to ensure activation of T cells only. Alternatively, for downstream experiments in which blocking the TCR might be an issue, cytokine activation is preferable, even though cells other than T cells might also be activated by this method, such as Natural Killer (NK) cells. In the experiments presented here, PBMCs were activated for 48 hours prior to the experiment, with either 30 U/mL IL-15 or 100 ng/mL OKT3. Non-activated PBMCs and spheroids cultured alone were used as controls. At the start of the experiment, 30,000 PBMC, activated or not were added to individual spheroids formed with the RCC7 cell line by the liquid overlay method as described above. After 24 hours of coculture, spheroids were thoroughly washed to remove non-infiltrated PBMCs remaining in the supernatant and the spheroids were dissociated using Accutase (see *Materials and Methods*). Infiltration was then measured by flow cytometry, using CD45 positivity as a marker of infiltrating leukocytes. The results were analyzed to show the percentage of CD45+ cells within the total cells obtained from the dissociation.

**Figure 4:**
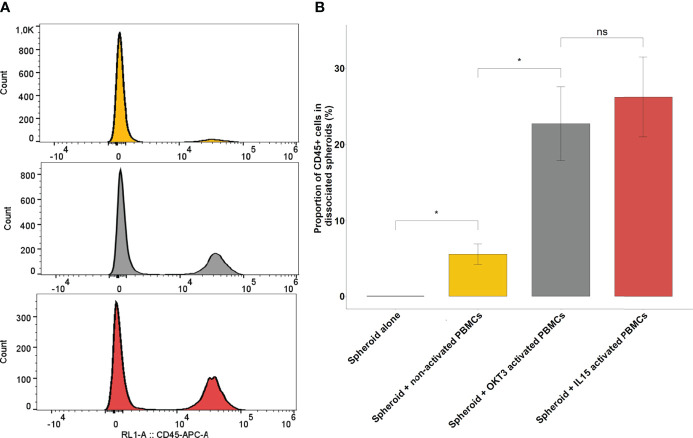
Immune infiltration following co-culture between RCC spheroids and activated or non-activated PBMCs. **(A)** Representative CD45 flow cytometry histograms of 3 pooled, washed and dissociated spheroids after 24h of culture, alone, in co-culture with non-activated PBMCs or with PBMCs activated with IL-15. **(B)** Barplots representing the percentage of CD45 positive cells found by flow cytometry in the dissociated spheroid after co-culture in the indicated conditions of activation. Results for a representative experiment out of 3, performed in quadruplicates. Error bars represent standard error, statistical significance (p<0.05) of the Student test is notified by a star. *p < 0.05; ns, non-significant.

**Figure 4A** shows representative examples of the results obtained when non-activated and IL-15-activated PBMCs were used to infiltrate spheroids. As can be seen, even though some non-activated PBMCs could infiltrate the spheroids, activation yielded a much more significant infiltration.

**Figure 4B** shows the extent of the infiltration obtained from one representative experiment out of five performed for this report, with all conditions done in quadruplicate. In non-activated conditions, infiltrated PBMCs represented only 5.52% ± 1.33 of the overall population of cells obtained after spheroid dissociation. By contrast, OKT3-activated and IL-15-activated PBMCs represented respectively 22.8% ± 4.84 and 26.2% ± 5.2 of cells obtained after spheroid dissociation

Both OKT3 and IL-15 can be used to activate PBMCs and induce spheroid activation. The choice between the two methods depends on the downstream experiment: if TCR implication is not an issue, either can be used, whereas if TCR interaction is required, IL-15 would be preferable. In the next set of experiments, since TCR interaction with tumor HLA is required, IL-15 activation was used.

Monocytes can also infiltrate spheroids:after isolation from donor’s PBMCs, 30,000 monocyte derived cells were cocultured with 10,000-cell RCC7 formed spheroids. After 24 hours, the spheroids were washed and dissociated as described above. The obtained cells were characterized by flow cytometry, looking for CD14 expressing cells and HLA-DR expressing cells to assess myeloid monocyte derived antigen presenting cells infiltration.

**Figure 5:**
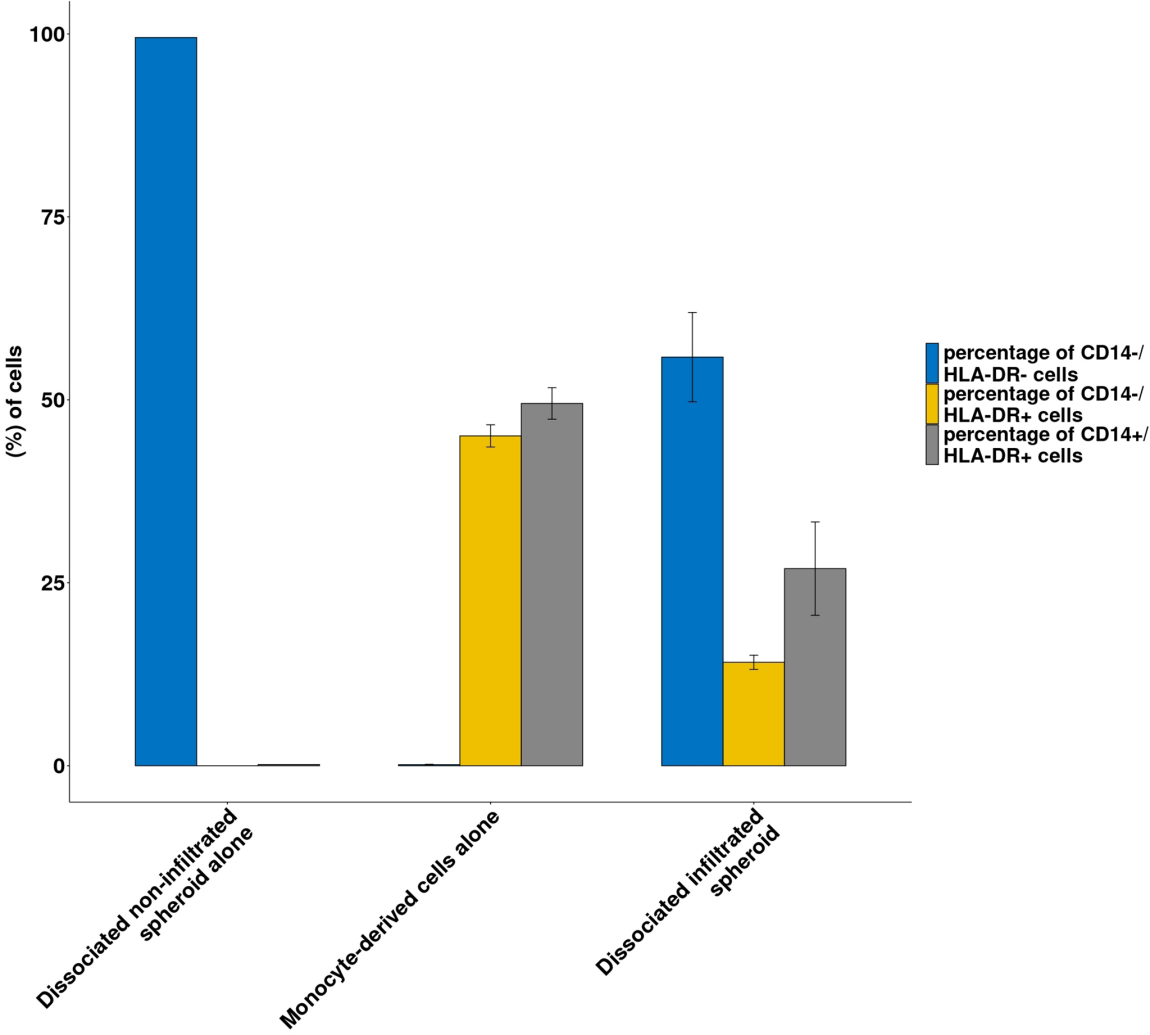
Immune infiltration following co-culture between RCC spheroids and monocyte-derived cells. Histograms representing the proportion of CD14-/HLA-DR- cells (spheroid cells) or CD14+/HLA-DR^+^ and CD14^-^/HLA-DR^+^ from the monocyte-derived population in the dissociated spheroid cultured alone or monocyte derived cells cultured alone or dissociated infiltrated spheroids are shown Results from a representative experiment; N=3; all conditions done in triplicate. Means ± SE are represented.

**Figure 5** shows the proportion of CD14+/HLA-DR+, CD14-/HLA-DR+, CD14-/HLA-DR- in controls (monocyte-derived cells alone or dissociated non-infiltrated spheroids alone) and in dissociated infiltrated spheroids.

As shown in **Figure 5**, spheroids cultured alone contained no CD14 or HLA-DR expressing cells while monocyte-derived cells contained 96% of HLA-DR positive cells. By contrast, dissociated spheroids that had been cocultured with monocyte-derived cells contained 41% HLA-DR+ cells. Interestingly, while the original HLA-DR positive monocyte-derived cells contained roughly equal amounts of CD14- and CD14+ cells, spheroid-infiltrating monocyte derived cells contained 2/3 CD14+ cells and 1/3 CD14- cells.

Thus spheroid can be infiltrated by several cell types according to the specific needs of a subject of study and can therefore constitute an immunocompetent *in-vitro* model for studies in immuno-oncology.

### Live Imaging Spheroid Killing Assay for the Assessment of Immune Checkpoint Blockade Efficacy

One of the possible and most explored applications of spheroids is to serve as a preclinical model for drug efficacy assessment. Thus, as an example of an application for an infiltrated spheroid model, we chose to implement a live spheroid immune killing assay. In this type of assay, immune cells are expected to cause spheroid destruction by exerting cytotoxicity. In the experiments presented here, spheroids are generated with PD-L1-positive RCC7 cells, effector cells are IL-15-activated allogeneic PBMCs, and treatments are 2 therapeutic anti-PD-1 antibodies: nivolumab and pembrolizumab. Both these checkpoint inhibitors are currently used as a treatment for metastatic ccRCC. In this experiment, it is expected that PD-L1 expressed by spheroid cells will interact with PD-1 expressed by activated cytotoxic cells, reducing their killing capabilities, and that nivolumab and pembrolizumab anti-PD-1 antibodies will block PD-1-PD-L1 interaction and increase tumor cell destruction.

All procedures for the experiment are detailed in the Materials and Methods section. Briefly, after 48 hours of IL-15 activation, treatments (10 µg/mL of either nivolumab or pembrolizumab) were added to the wells containing the PBMCs, leaving non-treated wells as control. In order to ensure spheroid killing, we used 100,000 PBMCs per well (10:1 effector:target ratio). They were then added to the wells of a 96-well cell-repellent plate containing 2-day old spheroids composed of 10,000 RCC7 cells. Wells with either no PBMCs, or non-activated PBMCs served as control for each condition. Finally, 5 µg/mL of Propidium Iodide were added to track cell death. Using the Incucyte S2 system, each well was imaged for brightfield and red fluorescence every 2 hours for five days.

**Figure 6:**
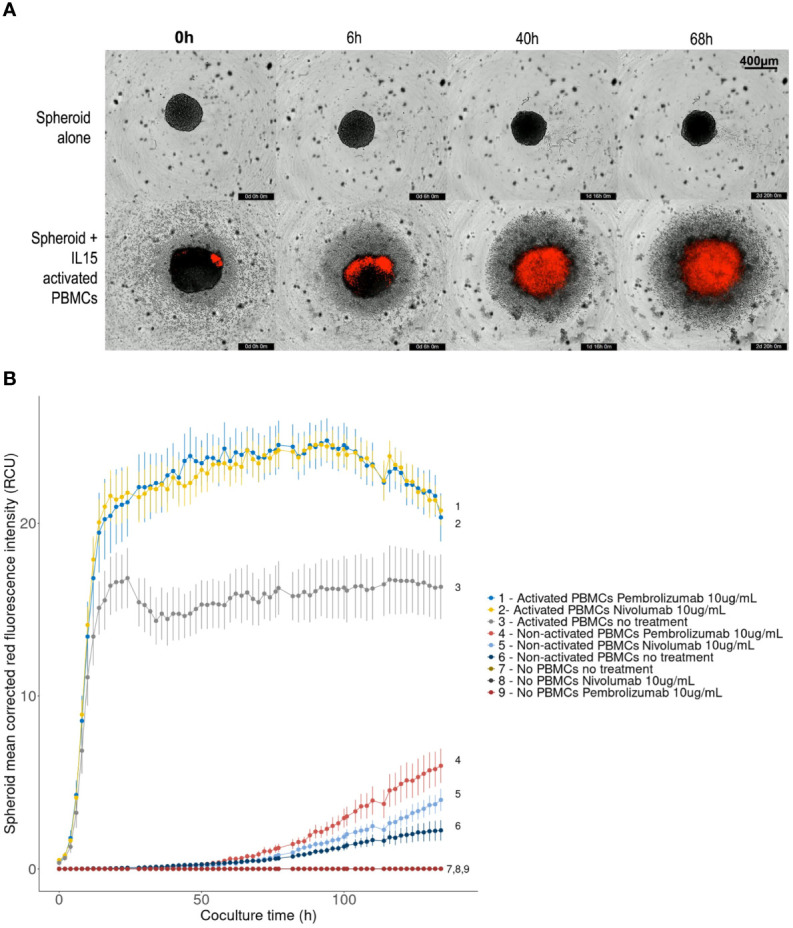
Effect of PD-1 blockade on spheroid destruction by IL-15 activated allogeneic PBMCs. 10,000-cell RCC7 spheroids were cultured alone or co-cultured with activated or non-activated PBMCs for 5 days, in the presence or absence of nivolumab or pembrolizumab. The wells were imaged every 2 hours with the Incucyte S2 system. Propidium Iodide staining was used to track cell death. **(A)** Representative 4× brightfield images with superimposed red fluorescence (Propidium Iodide), background level was set by fixing control spheroid red fluorescence to zero. **(B)** Linechart of spheroid red fluorescence measurement (Red Calibrated Unit) over time as calculated by the Incucyte S2 system in the given conditions. Means of 10-plicates, representative results out of 3 experiments. Error bars represent the Standard Error of the means, digits correspond to conditions.

Brightfield + red fluorescence live images presented in **Figure 6A** show two key parameters: spheroid disruption and tumor cell death, on non-treated spheroids. Spheroids cocultured with IL-15-activated PBMCs started disaggregating after 24h compared to control spheroids cultured alone.

For immune-cell induced tumor cell death measurement using PI fluorescence, red-fluorescence was adjusted so that background from control spheroids was null. After fluorescence background removal, increasing levels of PI red fluorescence were observed in cocultured spheroids, indicating cell death throughout the experiment (**Figure 6A**), implying that activated PBMCs lead to spheroid disruption and killing.

**Figure 6B** presents the mean Propidium Iodide red-fluorescence of the spheroids calculated by the Incucyte system during the five-day experiment. An increase in red fluorescence means an increase in cell death. As shown, coculturing spheroids with allogeneic PBMCs, activated or not, lead to increased levels of red fluorescence indicating increased spheroid cell death. However, regardless of treatment, this effect remained limited with non-activated PBMCs: fluorescence started increasing after 50 hours and reached 2-6 Red Calibrated Unit (RCU) at day 5, whereas with IL-15-activated PBMCs, fluorescence increased sharply to plateau in 16-20 hours. Significantly higher levels of cell death were reached when treating PBMCs with nivolumab or pembrolizumab. The largest difference was measured after 36 hours when red fluorescence reached 23 RCU in presence of nivolumab or pembrolizumab, representing a 40% increase compared to non-treated controls (16 RCU). No differences between the effects of nivolumab or pembrolizumab were observed.

In this type of experiment, the most favorable timeframe for observing the effect of the immunotherapeutic compounds was therefore between 16 and 84 hours. Videos for each condition can be found in **Supplementary Data**.

## Discussion

It is now well-established that the spheroid model can provide relevant insight on cancer biology while remaining relatively simple and straightforward. Its ability to recapitulate key tumor characteristics has been extensively studied in several cancers. A particularly relevant feature is its ability to conserve a certain amount of complexity from the original tumor. ccRCC has long been known as a cancer of high heterogeneity, both in terms of tumor cell clonality and TME characteristics and there is increasing evidence of the role of tumor heterogeneity in treatment resistance and disease progression (32–34). ccRCC could therefore benefit from the use of the spheroid model. In particular, patient-derived spheroids as described in this work retain a fraction of the original tumor complexity, and allow culturing these heterogeneous cells in close contact, offering possibilities for enhanced cell-to-cell interaction as compared to 2D models.

While the spheroid model appears to take an increasingly important place in cancer research, there are very few occurrences of its application to ccRCC. Remarkably, Rausch et al. described a model of infiltrated ccRCC spheroid composed of controlled proportions of cancer cells, fibroblasts, endothelial cells and immune cells subsets, all from established cell lines (13). In light of this work, we were able to confirm the infiltration of different subsets of immune cells in ccRCC spheroids and to complete the methods used for the formation of spheroids with patient-derived spheroids.

The aim of this study was to present standardized methods to constitute a simple and adaptable toolbox to establish ccRCC spheroid models. It is addressed to researchers wishing to initiate studies relying on the spheroid model. We introduced methods and a new medium for cultivating primary ccRCC cells and forming spheroids, from both established cell lines and primary ccRCC cell cultures. We proposed a method to improve the reliability of the data obtained in this model by selecting the spheroids for subsequent experiments based on morphological criteria. We showed that the spheroids can be infiltrated by activated immune cells, resulting in a 3D model suitable for cancer immunology studies. As an example, we implemented a live-imaging spheroid killing assay showing the effect of PD-1 blockade on spheroid destruction by activated allogeneic immune cells.

This method is highly adaptable and changes can be made if necessary: the size of the spheroids can easily be adapted by changing the number of cells used to form the spheroid and other elements can be mixed in the cell suspension before the formation of the spheroid as it has been done before with stromal or endothelial element (30).

Because they are harder to handle than 2D models, spheroids can only be used in small numbers during studies. Thus, reproducibility and representativity and therefore parallelization can be an issue (12, 35). As shown, morphological criteria measured on routinely taken images can help limit this heterogeneity by selecting a homogeneous population of spheroids before subsequent experiments and thus enhance the relevance of the data obtained. Here, a Fiji macro permitted fast and semi-automatized spheroid quality analysis.

The methods presented here allow for the formation of heterotypic spheroids. However, it remains to be seen if the spheroids generated following these methods are representative of the original tumor complexity. Indeed, there are several key steps that could lead to a loss in representativity. First, complete tumor dissociation leads to the loss of original structure. A different approach to forming spheroids that partially retain this structural information has been carried out before by Jenkins et al. on different cancer types (36). These “Patient-Derived Organotypic Tumor Spheroids” were formed by partial tumor digestion until the desired size is reached. While this approach retains a form of the original tumor structure, it raises reproducibility issues as each formed spheroid contains specific elements not shared among all other spheroids, therefore hindering parallelization. Secondly, to reach maximum representativity, it would be preferable to avoid any 2D culture step and form spheroids directly after tumor dissociation to retain a maximum of tumor elements. Indeed, it is very likely that the amplification step carried out in plastic flasks leads to the selection of certain tumor elements, based on their capacity to adhere to plastic. Complementary studies on the representativity of patient-derived ccRCC spheroids such as transcriptomic studies would provide precious data on the level of complexity recapitulated by this model, along with their resemblance with the original tumor. This is critically important if the final aim is to test therapeutic molecules in the context of personalized medicine, particularly in the case of checkpoint inhibitors as checkpoint molecule expression may vary during cultures. To conclude, forming patient-derived ccRCC spheroids can be done using simple techniques. Further studies to assess the preservation of tumor complexity and improve the assessment of cell interactions within this model are ongoing to validate its relevance, both as a translational research tool but also as a candidate theranostics assay.

## Data Availability Statement

The raw data supporting the conclusions of this article will be made available by the authors, without undue reservation.

## Author Contributions

LL conceived and designed the analysis, collected the data, performed the analysis and wrote the manuscript. GM conceived, designed the analysis, collected the data, performed the analysis and wrote the manuscript. RL collected the data and performed the analysis. CD wrote the manuscript. FB provided tumor samples. MD provided tumor samples, AM-L provided tumor samples. FD provided tumor samples. EC conceived and designed the analysis and acknowledged the final version. NR-F conceived and designed the analysis, revised and acknowledged the final version. JL conceived and designed the analysis, wrote the manuscript, revised and acknowledged the final version. All authors contributed to the article and approved the submitted version.

## Funding

This study was funded by CEA. LL is recipient of a PhD grant through the CEA-focus program “organoids-on-a-chip”. GM was a recipient of a CEA PhD grant.

## Acknowledgments

We would like to thank the Saint-Louis Research Institute Core Facility and particularly Niclas Setterblad for his precious advice and kind help regarding imaging experiments.

## Conflict of Interest

The authors declare that the research was conducted in the absence of any commercial or financial relationships that could be construed as a potential conflict of interest.

## Publisher’s Note

All claims expressed in this article are solely those of the authors and do not necessarily represent those of their affiliated organizations, or those of the publisher, the editors and the reviewers. Any product that may be evaluated in this article, or claim that may be made by its manufacturer, is not guaranteed or endorsed by the publisher.
