# Supplementary material for: Methods for Establishing a Renal Cell Carcinoma Tumor Spheroid Model With Immune Infiltration for Immunotherapeutic Studies
Source: Front Oncol. 2022 Jul 28;12:898732. doi: 10.3389/fonc.2022.898732 (PMC9366089; doi:10.3389/fonc.2022.898732)
Supplement: Supplementary file 1 [file DataSheet_1.docx]

Renal Cell Carcinoma Dissociation Protocol

- Materials:

Solutions: DNase I (Sigma-Aldrich), Liberase TM (Sigma-Aldrich), HBSS containing Ca^2+^ and Mg^2+^, complete RPMI 1640 (Sigma), DMEM (Gibco), Ham’s F12, Red Blood Cell Lysis Solution (Miltenyi Biotech), PBS 1X

Protocol :

- Enzyme reconstitution:
- Dissolve the 5mg (26WU) of Liberase TM by adding 2mL of ultrapure water to the vial, aliquot by 134µL for use in 6mL at 0.28WU/mL.
- Working solution:
- Add 134µL of Liberase TM and 30µL DNase I in 6mL of cold HBSS, in a 15mL conical tube. Keep on ice.
- Sample preparation:
- Carefully rinse the samples with PBS 1X.
- In a petri dish, using 2 scalpels, discard the fibrous parts, and finely mince the samples, until reaching approximately 1mm^3^ pieces.
- Dissociation:
- Transfer the pieces to the falcon containing the working solution and incubate for 30-40 minutes in a water bath set at 37°C.
- Shake the tubes every 10 minutes by hand, strenuously but carefully not to damage the cells. No vortexing or trituration using up-and-down pipetting should be involved.
- At the end of dissociation, no remaining tissue should be visible in the tubes, except if fibrous parts were remaining.
- After 40 minutes maximum, resuspend the dissociate by hand-agitation before running it through a 100µm sieve on top of a 50mL conical tube.
- Pour 40mL of cold RMPI containing 10% FCS on the sieve to stop the dissociation. Centrifuge at 300g for 10 minutes and aspirate supernatant completely.

From there, if necessary, tumor cells and TILs can be separated using a CD45 magnetic beads sorting (Miltenyi). This step requires performing a red blood cell lysis step beforehand (Red Blood Cell Lysis Solution) (Miltenyi).

Renal Cell Carcinoma Spheroid Formation Protocol

- Materials:

Solutions: Renal Cell Carcinoma Culture Medium (RCCM): DMEM/F12 3:1 (v:v) + 10% SVF + 2 mM L-glutamine + 100 U/ml penicillin/streptomycin + Epithelial Growth Factor (10ng/mL) + 0.4 μg/ml hydrocortisone (0.4µg/mL), Trypsin-EDTA

Resource: 96-well U-bottom cell-repellent plate

- Protocol:
- Culture the desired RCC cells (patient-derived or cell line) until reaching 80% confluence.
- Gently detach the cells using trypsin.
- Wash the cell in complete medium and resuspend in RCCM at desired concentration

*ex: for 10,000 cells spheroids, cells should be at 50,000C/mL, 500-20,000 cells/spheroids work best*

- Distribute 200µL of suspension per well
- Centrifuge the plate at 500g for 1 minute
- Culture in standard conditions and monitor the formation each day
- Tips on spheroid handling:
- Spheroids fall rapidly to the bottom of the wells, no centrifugation is needed
- Placing a dark surface underneath the plate helps seeing the spheroids
- Spheroids are easily aspirated in 200µL pipets, put the tip right on top of the spheroid.

Spheroid Immune Infiltration

- Materials:

Solutions: human IL-15, complete RPMI 1640 medium, formed spheroids in their cell-repellent plate (see above)

- Protocol:
- 2 days prior to infiltration, activate the immune cells (PBMCs or TILs) as follows:
  - Distribute the number of desired immune cells in a 96-well standard U-bottom plate in complete RPMI, final volume: 200µL.
  - Add 40U/mL IL15 and culture in standard conditions for 48 hours
- Experimentation day:
  - Remove the supernatant in the spheroid plate wells without aspirating the spheroids
  - Transfer the immune cells to the spheroid plate wells
- Functional studies:
  - If relevant, treatments acting on immune cells must be added before transferring to the spheroids
  - Infiltrated spheroids can be analyzed in flow cytometry after dissociation (see Grässer et al. 2018), avoid too many washing steps, perform washes in Eppendorf tubes and pool spheroids to obtain a sufficient number of cells for flow-cytometry

Live Imaging Spheroid Killing Assay

- Materials:

Solutions: Propidium Iodide, (treatments)

Resource: formed spheroids, activated immune cells

- Protocol
  - 2 days prior to experiment, activate immune cells and form spheroids (see above)
  - On day 0, treat the immune cells or the spheroids with relevant treatment
  - Add 5µg/mL Propidium Iodide to track cell death
  - Image using an Incucyte S2 system, every 2 hours, 4x images brightfield + phase-contrast + red fluorescence + green fluorescence.
  - Analysis can be done as follows:
    - Mode : spheroid
    - Measurement “Largest Object mean red fluorescence intensity
    - The ratio between background and cells for spheroid detection can be set on minimum (favorable to background)
    - A minimum object size can be set at 10,000 to avoid misdetection of the spheroid
  - Background fluorescence correction:
    - A fair amount of background fluorescence can be observed as PI penetrate the cells over time even when not dead.
    - It can be corrected by augmenting the “green to red percentage” until the control spheroids (cultured without PBMCs and without treatment) show no sign of red fluorescence on images.
