# Supplementary material for: Methods for Establishing a Renal Cell Carcinoma Tumor Spheroid Model With Immune Infiltration for Immunotherapeutic Studies
Source: Front Oncol. 2022 Jul 28;12:898732. doi: 10.3389/fonc.2022.898732 (PMC9366089; doi:10.3389/fonc.2022.898732)
Supplement: Supplementary file 3 [file Image_1.pdf]

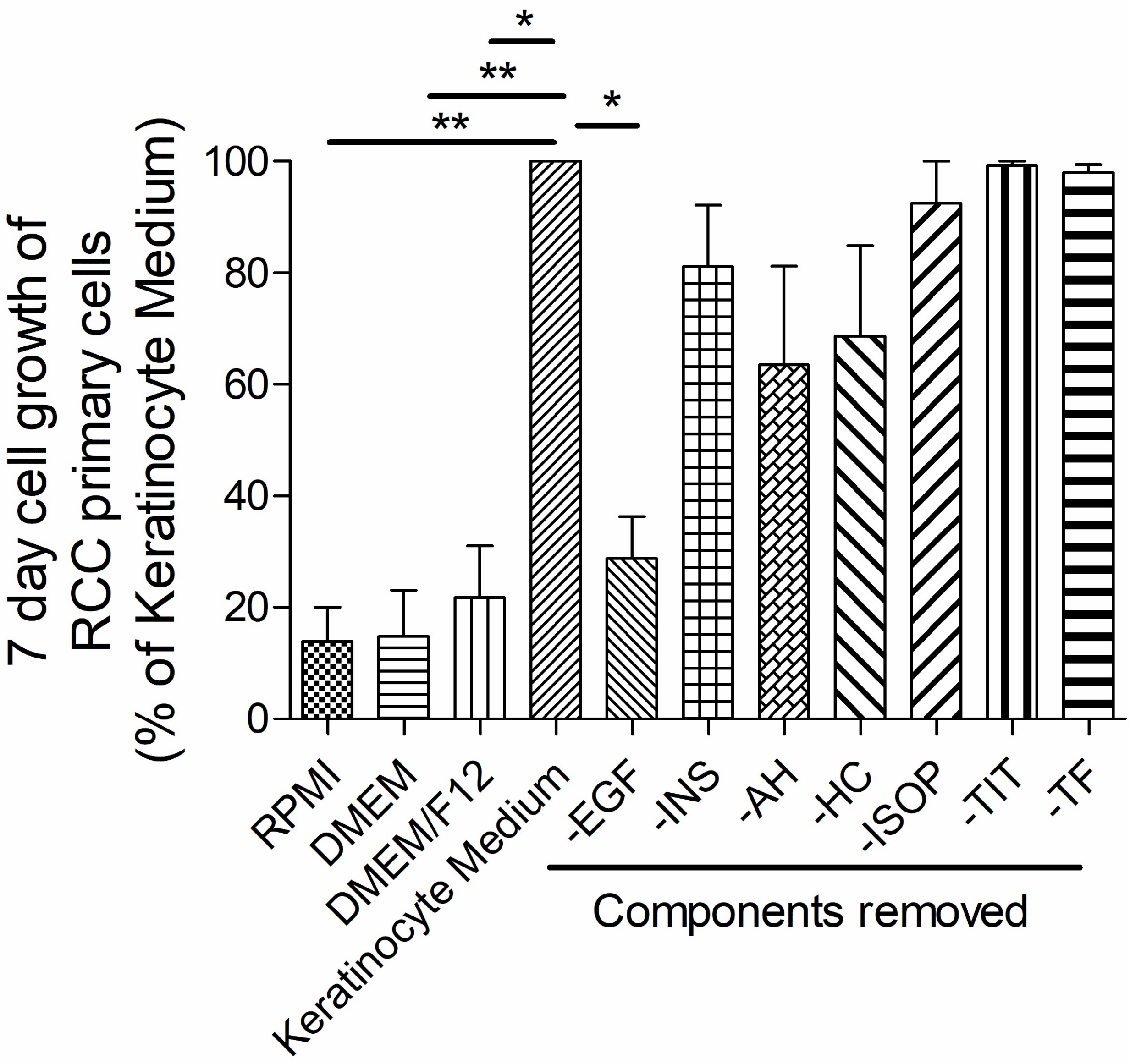

Supplementary Figure 1. Determination of the components responsible for increased RCC cell growth in a keratinocyte medium. 7-day proliferation index of ccRCC cells from 3 patients in commercial media (RPMI, DMEM, DMEM/F12), in a keratinocyte medium composed of DMEM/F12 + Epithelial Growth Factor (EGF) + Insulin (INS) + Adenin (AH) + Hydrocortisone (HC) + Isopropanol (ISOP) + Triodothyronine (TIT) + Transferrin (TF) or in keratinocyte medium missing one component (–component). Maximum proliferation was obtained with keratinocyte medium and constituted the 100% mark. All other proliferation results were indexed on this mark (mean+SEM, $p<0,05$, $n=3$). Exact p-values were determined according to the paired t-test. * : $p<0.05$ and ** : $p<0.01$.
