# Supplementary material for: Methods for Establishing a Renal Cell Carcinoma Tumor Spheroid Model With Immune Infiltration for Immunotherapeutic Studies
Source: Front Oncol. 2022 Jul 28;12:898732. doi: 10.3389/fonc.2022.898732 (PMC9366089; doi:10.3389/fonc.2022.898732)
Supplement: Supplementary file 4 [file Table_1.docx]

**Supplementary Table 1.** Specific materials used for RCC culture, dissociation and spheroid formation and PBMC activation

| REAGENT or RESOURCE | SOURCE | IDENTIFIER |
| --- | --- | --- |
| *Renal Cell Carcinoma Culture Medium* |  |  |
| DMEM | Gibco | 10566016 |
| F12 | Gibco | 11765054 |
| EGF | Merck Millipore / Sigma-Aldrich | GF144 |
| Hydrocortisone | Sigma-Aldrich | H4881 |
| *Dissociation* |  |  |
| Liberase TM | Sigma-Aldrich | 5401119001 |
| DNase I | Sigma-Aldrich | 04716728001 |
| HBSS Ca^2+^ Mg^2+^ | Gibco | 11560616 |
| Red blood cell lysis solution | Miltenyi Biotec | 130-094-183 |
| CD45 MicroBeads | Miltenyi Biotec | 130-045-801 |
| QuadroMacs separator | Miltenyi Biotec | 130-091-051 |
| *Spheroid formation* |  |  |
| Cell repellent 96-wells U-bottom plates | Greiner Bio-one | 650970 |
| *PBMC activation* |  |  |
| Human IL15 | Miltenyi Biotec | 130-095-764 |
| Purified anti human CD3 (OKT3) | Biolegend | 317302 |
